# An acutely ischaemic hand in a young adult due to subclavian artery aneurysm (SAA) caused by a cervical rib: A case report

**DOI:** 10.1186/1757-1626-1-140

**Published:** 2008-09-04

**Authors:** Karen Tam, Kanchana Seneviratne, Rajiv Pathak, Ajantha Jayatunga

**Affiliations:** 1 Vascular Surgical Department, Russells Hall Hospital, Dudley, UK

## Abstract

**Introduction:**

True subclavian artery aneurysms are relatively rare lesions. Thoracic outlet compression is responsible for 75% of aneurysms. They are formed as a result of compression by, for example a cervical rib.

**Case Presentation:**

We present a case of subclavian artery aneurysm secondary to a cervical rib in a 25-year-old young Asian adult, who presented with an acutely ischaemic upper limb. A Computed Tomography angiogram revealed a right sided cervical rib and sacular aneurysm in the mid-portion of the subclavian artery.

**Conclusion:**

Although it is a rare condition, it is important to be aware especially in the young age group. Surgical management has very favourable outcomes in these patients.

## Background

An aneurysm of the subclavian artery is uncommon. In the younger age group the cause is usually due to thoracic outlet compression often due to an extraneous cervical rib. One such exceptional case of arterial thoracic outlet syndrome presenting in a young man is described below.

## Case presentation

A, healthy 25 year old Asian male presented with a 3 day history of sudden onset worsening pain of the right forearm and hand. He was a non-smoker with no history of trauma and no past medical history of note. On examination, he had classical signs of acute limb ischaemia consisting of pallor, coldness with prolonged capillary refill of about 10 seconds and sensory and motor impairment. Pulsatile mass in the root of the right side of the neck with a bruit and flow murmur was also elicited. On hand held Doppler ultrasound of the right arm, a diminished ulnar artery signal and a monophasic radial artery signal were detected, along with a normal brachial artery signal. A clinical diagnosis of aneurysm of the subclavian artery was made with embolic occlusion of the brachial artery.

**Figure 1:**
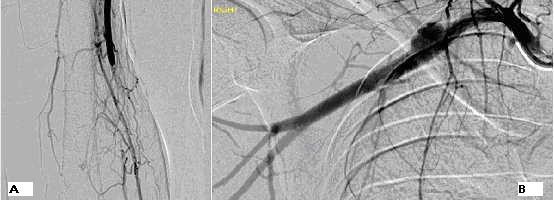
Right Brachial arteriogram (A) Occlusion of the brachial artery with an abrupt cut-off point. (B) Saccular aneurysm arising from the mid right subclavian artery; The first image (A) is of the Right brachial artery, its is an arteriogram and was taken a day after admission. Image (B) shows the right subclavian artery and its branches, it is also an arteriogram and was taken the day after admission.

At this point the patient was commenced on intravenous heparin and the symptoms partially improved. A brachial angiogram (Figure 1) and Computed Tomography angiogram revealed a right sided cervical rib and saccular aneurysm in the mid-portion of the subclavian artery, proximal to the clavicle. The aneurysm measured 1.1 × 2 cm. Brachial artery occlusion due to a thrombus extending from the mid-upper arm to the distal part of the elbow was also demonstrated. Intra-arterial thrombolysis was attempted but failed due to extravasation following direct puncture of the axillary artery. 6 days after initial presentation, the patient underwent a subclavian artery aneurysm repair with a cervical rib excision and brachial embolectomy. He was treated with warfarin for 3 months post-op. Post operative Doppler scan revealed good signals in the right ulnar and radial arteries. Follow-up of the patient in clinic 3 months post-discharge, revealed no complications and complete resolution of the symptoms.

## Discussion

True subclavian artery aneurysms are relatively rare lesions, accounting for approximately 0.13% of all aneurysms [1,2]. Atherosclerotic aneurysms are more commonly found in males over the age of 60 years [3,4]. Thoracic outlet compression is responsible for 75% of aneurysms which arise from the distal part of the subclavian artery, also known as extrathoracic subclavian artery or subclavian-axillary artery aneurysms [3]. They are formed as a result of compression by, for example a cervical rib, malalignment post-clavicular fracture or congenital bands on the artery as it passes through the interscalene triangle. This causes stenosis and angulation of the artery, which with time will lead to post-stenotic dilatations [4,5]. However, these aneurysms are only found in 1.1% of patients with thoracic outlet syndrome (TOS) [6]. Patients may present with symptoms of thromboembolism, such as digital ischaemia, Raynaud's phenomena, pulsatile masses in the supraclavicular fossa or axilla, brachial plexopathy or Horner's syndrome [4].

The remainder of the aneurysms arise from the proximal part of the subclavian artery, also known as intrathoracic or proximal subclavian artery aneurysms [3]. They are mainly the result of atherosclerosis and are usually asymptomatic [3]. However, symptoms of compression, including discomfort in the neck, dysphagia and dyspnoea have been reported. Other causes of subclavian artery aneurysms include trauma, iatrogenic; and rarely arteritis, cystic medial necrosis, congenital, syphilis and tuberculosis [4,7].

Plain films and ultrasound scanning are used to identify bony abnormalities and confirm the presence of an aneurysm. However CT or magnetic resonance imaging angiography remain the investigations of choice [3,4].

Open surgical repair is the standard management, even for asymptomatic subclavian artery aneurysms due to their tendency to increase in size or rupture [7]. This involves resection of the aneurysm and reconstruction of the artery with or without graft insertion. Decompression of the TOS with resection of the cervical and/or 1^st ^rib, anterior scalene muscle or constricting fibrous bands; or median sternotomy and lateral thoracotomy may be necessary for intrathoracic aneurysms [4,7]. This depends on the cause and site of the aneurysm. Distal arterial occlusion secondary to emboli will warrant angioplasty or thromboembolectomy to restore patency of the vessels [3]. Overall, surgical management has very favourable outcomes in these patients [4].

## Conclusion

Cervical rib leading to subclavian artery aneurysms should always be included in the differential diagnosis of an acutely ischaemic hand. It is a rare event especially in the young adult. This report illustrates the importance and difficulty of correctly diagnosing a rare pathology that has a common presentation. It is important to recognise subclavian artery aneurysms secondary to cervical rib in patients with TOS as they can be safely managed preventing rupture and further thrombosis.

## Consent

Written informed consent was obtained from the patient for publication of this case report and accompanying images. A copy of the written consent is available for review by the Editor-in-Chief of this journal.

## Competing interests

The authors declare that they have no competing interests.

## Authors' contributions

KT and KS undertook writing, literature review, manuscript preparation and literature search. KS submitted the article. RP was responsible for diagnosis, patient management and review. KT and KS managed the patient on the ward. AJ reviewed the final manuscript. All authors read and approved the final manuscript.
